# Investigation of the Transient Behavior of a Cantilever Beam Using PVDF Sensors

**DOI:** 10.3390/s120202088

**Published:** 2012-02-14

**Authors:** Chien-Ching Ma, Yu-Hsi Huang, Shan-Ying Pan

**Affiliations:** 1 Department of Mechanical Engineering, National Taiwan University, Taipei 106, Taiwan; E-Mail: r96522508@ntu.edu.tw; 2 Graduate Institute of Applied Science and Technology, National Taiwan University of Science and Technology, Taipei 106, Taiwan; E-Mail: yhhuang@mail.ntust.edu.tw

**Keywords:** PVDF film sensor, strain gage, cantilever beam, transient response, resonant frequency

## Abstract

In this paper, a PVDF film sensor was used to measure the transient responses of a cantilever beam subjected to an impact loading. The measurement capability of a PVDF sensor is affected by the area of the PVDF film sensor and the signal conditioner (charge amplifier). The influences of these effects on the experimental measurements were investigated. The transient responses for the dynamic strain of the beam were measured simultaneously by the PVDF sensor and a conventional strain gauge. The resonant frequencies of the beam were determined by applying the Fast Fourier Transform on transient results in the time domain of the PVDF sensor and the strain gauge. The experimentally measured resonant frequencies from the PVDF sensor and the strain gauge were compared with those predicted from theoretical and FEM numerical calculations. Based on the comparison of the results measured for these two sensors, the PVDF film sensor proved capable of measuring transient responses for dynamic strain, and its sensitivity is better than that of the strain gauge. Furthermore, almost all the resonant frequencies can be obtained from the results of transient responses for PVDF film.

## 1. Introduction

The phenomenon of polymorphism of polyvinylidene fluoride (PVDF) has been investigated since the 1960s [1,2]. Four structures of PVDF were observed, and the three common notations used for indicating these structures are {I, II, III, IV}, {β, α, γ, δ}, and {I, II, III, II_p_} [3,4]. It was the β phase (phase I) of PVDF, found by Kawai in 1969 [5], that exhibited the strongest piezoelectric activity of any known polymer. A variety of methods like mechanical stretching [6], application of an electric field [7] or incorporation of additives [8,9] has been used in the literature to obtain this crystalline phase. PVDF materials are produced in the form of thin films with thicknesses ranging from 9 to 110 μm. In addition, 0.5- and 1-mm thick films are commercially available. Recently, the spin-coating method [10,11] has been reported for obtaining thin films of β-PVDF. This technique allows the fabrication over large area on the substrates. High-quality films with controlled thicknesses from 300 nm to 4.5 μm can be obtained in a single deposition step using the spin-coating method [12].

The electrodes are produced by sputtering iron, cobalt, nickel, or aluminum particles on the surfaces of PVDF. Like other piezoelectric materials, PVDF has piezoelectric effects. When PVDF is pressed or stretched, electrons are charged on the electrodes. Therefore, electric signals are generated that are related to the pressure and stretch that is applied to PVDF. On the other hand, when PVDF is placed in an electrical field, it is deformed. This PVDF piezoelectric polymer has the most significant piezoelectricity among all piezoelectric polymers. In addition, piezoelectric polymers have higher flexibility and mechanical strength than other piezoelectric materials. The applications of PVDF sensors include the realms of underwater investigation, biomedical studies, nondestructive damage detection, robotics, and vibration control.

PVDF has been investigated intensively since 1969. An enormous amount of research has investigated the characteristics of PVDF [13–19]. The acoustic impedance of PVDF (about 3.94 × 10^6^ rayl) is close to that of water (about 1.5 × 10^6^ rayl) [20], hence it can be used in the water environments without matching layers. This special characteristic makes PVDF suitable for underwater investigation, underwater acoustics, and biomedical transducers [21–24]. Due to its low weight, high flexibility and high mechanical strength, PVDF can be easily attached to surfaces of structures without influencing the resonant frequencies of those structures. PVDF was also utilized in the realm of nondestructive damage detection [25–30], and the first case was presented by the USA Naval Air Development Center. The Center used PVDF sensors to detect flaws and structural defects in aircrafts [21,31]. Furthermore, PVDF can be used as a pressure sensor, tactile sensor, glide sensor, and temperature sensor for mechatronics [32]. The responses of PVDF under static and cyclic loading conditions and the significance of cyclic frequency and mean stress have been studied in terms of time-dependent mechanical responses [33,34]. Based on these experimental results, a constitutive model of PVDF has been developed.

In this paper, the principles behind the PVDF sensing system and the vibration of a cantilever beam are briefly presented in Sections 2 and 3, respectively. Then, in Section 4, dynamic strain measured by the PVDF sensor and the strain gauge is presented. In addition, the influence of the size of the PVDF film and the presence of a charge amplifier on sensing ability is presented. All of the results obtained with a PVDF sensor were compared to those obtained with a strain gauge, as well as theoretical predictions and FEM results.

The sensitivity and accuracy of PVDF sensors presented in this study demonstrate their excellent characteristics in measuring dynamic strain in transient situations. The PVDF sensors are capable to obtain the transient responses of structures due to small impact loadings. The high signal to noise ration of PVDF sensors make them much more attractive in situations of low strain or high noise level. It is demonstrated in this study that the resonant frequencies can be easily and accurately determined from the measured transient responses. Almost all the resonant frequencies, which includes bending and torsional modes, can be obtained from larger size of the PVDF sensors. Furthermore, the influence of the locations of the sensors and impact loadings on the frequency spectrum is also discussed in detail.

## 2. PVDF Sensing System

### 2.1. PVDF Sensing Principle

**Figure 1.**
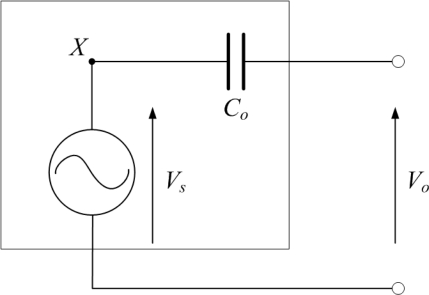
The electrical model of the PVDF film sensor.

PVDF film sensors comprise one type of capacitive sensors. The electrical model of a PVDF film sensor is shown in Figure 1, in which *C*_0_ indicates the equivalent capacitance of the PVDF film, *V_o_* is open-circuit voltage of the PVDF film, and *V_s_* can be considered to be an ideal voltage generator.

**Figure 2.**
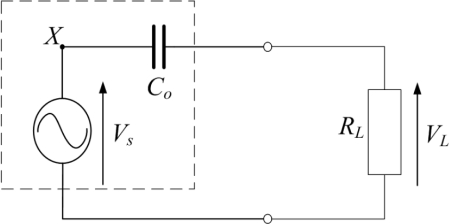
A PVDF sensor connected to an oscilloscope.

The equivalent capacitance *C*_0_ is expressed as [35]:

(1) $$C_{0}=\varepsilon \frac{A}{t}$$

where *A* is the area of the PVDF film covered by electrodes, *t* is the thickness of the PVDF film, and *ε* is the permittivity of the PVDF film. A PVDF sensor connected to an oscilloscope is shown in Figure 2, where *R_L_* is the input resistance of the oscilloscope, *Z_c_* is the equivalent source impedance of the PVDF film, and *V_L_* is the output voltage measured by the oscilloscope. The source impedance combined with the input resistance produces a voltage divider; hence, *V_L_* is expressed as:

(2) $$V_{L}=V_{s}\;\frac{R_{L}}{R_{L}+Z_{c}}$$

in which *Z_c_* is expressed as:

(3) $$Z_{c}=\frac{1}{j\omega C_{0}}$$

where $j=\sqrt{-1}$ and *ω* is the angular frequency or angular speed (measured in radians per second). The input resistance affects low-frequency measurement capability and signal amplitude. This is called the “loading effect”. As the ratio of input resistance to source impedance decreases, the overall output voltage is reduced.

The magnitude of the voltage, |*V_L_*|, is expressed as:

(4) $$\left|V_{L}\right|=\frac{\left(g_{3n}\;X_{n}\;t)(R_{L}\;C_{0}\;\omega \right)}{R_{L}^{2}\;C_{0}^{2}\;\omega^{2}+1}\;\sqrt{R_{L}^{2}\;C_{0}^{2}\;\omega^{2}+1}$$

where *g*_3*n*_ is the piezoelectric coefficient in *n* direction of the PVDF film and *X_n_* is the stress in *n* direction. The phase of the voltage, *ϕ* is indicated as:

(5) $$\phi ={\text{tan}}^{-1}\frac{1}{R_{L}\omega C_{0}}$$

**Figure 3.**
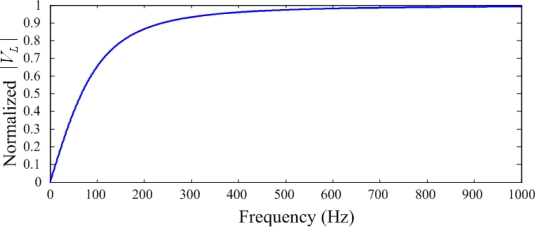
Amplitude spectrum of a PVDF sensor.

The relationship between normalized |*V_L_*| and frequency (Hz) is shown in Figure 3, where *t* = 28 μm, *A* = 300 mm^2^, *ε* = 107 × 10^−12^ (*F/m*) and *R_L_* = 1 MΩ.

**Figure 4.**
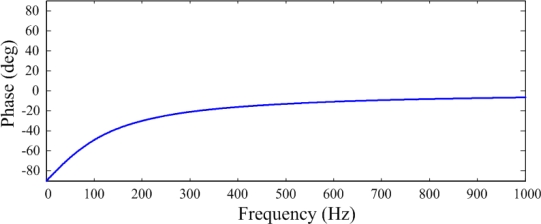
Phase spectrum of a PVDF sensor.

The relationship between *ϕ* and frequency (Hz) is shown in Figure 4. These two figures indicate that a capacitive sensor has a similar characteristic to a high-pass filter. The phase and the magnitude of the voltage are related to the frequency at low frequencies. The cut-off frequency is expressed as:

(6) $$f_{c}=\frac{1}{2\pi R_{L}C_{0}}$$

Below the cut-off frequency, the measured signals are proportional to the variation of strain; above the cut-off frequency, the measured signals are proportional to strain. Equation (6) indicates that the cut-off frequency is related to the properties of the PVDF sensor and the input resistance. Choosing a proper input resistance for the electronic interface is important in minimizing the loading effect. We used a signal conditioner as the intermediary between the PVDF film sensor and the oscilloscope.

The signal conditioner plays a crucial role in the measurement, and it can affect the performance and precision of the measuring system. Signal conditioners are bridges between sensors and other instruments. All moderate processes of obtaining the measured signal including amplifying, filtering, linearizing, and normalizing processes are called signal-conditioning processes. To make the measured signal appropriate to the post processes, these processes are performed properly to moderate measured signals. The signal conditioner used in this study is the 2775AM4 signal conditioner manufactured by Endevco.

**Figure 5.**
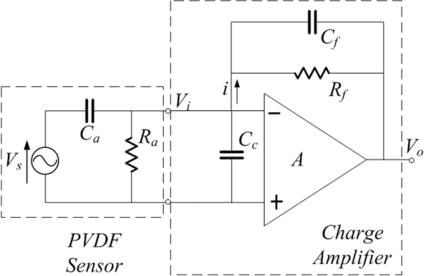
Equivalent-electric model of a PVDF sensor and charge amplifier.

A PVDF film sensor is a self-generated sensor, which means that it does not need to be linked with any power generator. The signal conditioner for a PVDF film sensor is a charge amplifier that can transfer charge signals that accumulate on electrodes of a PVDF sensor into voltage signals. A system consisting of a PVDF sensor and a charge amplifier is shown in Figure 5.

The left side of the figure shows an equivalent-circuit model of a PVDF sensor, whereas the right side shows an equivalent-circuit model of a charge amplifier [17]. In this system, *V_s_* is the voltage generated by the PVDF sensor; *R_a_* is the output impedance of the PVDF sensor; *C_a_* is the equivalent capacitance of the PVDF sensor; *C_c_* is the equivalent capacitance of electric wire; *V_i_* is the input voltage of the charge amplifier; *C_f_* and *R_f_* are the feedback capacitance and impedance of the charge amplifier, respectively; *A* is the gain of the charge amplifier; and *V_o_* is the output voltage of the charge amplifier. The output voltage is expressed as:

(7) $$V_{o}=\frac{-j\omega AV_{s}\;C_{a}}{\left[j\omega \left((A+1)C_{f}+C_{a}+C_{c}\right)\right]+\left[\frac{1}{R_{a}}+(A+1)\frac{1}{R_{f}}\right]}$$

If the gain of the charge amplifier is large enough, Equation (7) can be simplified as:

(8) $$V_{o}=\frac{-j\omega V_{s}\;C_{a}}{j\omega C_{f}+\frac{1}{R_{f}}}$$

In the high frequency region, the term $\frac{1}{R_{f}}$ can be neglected. Therefore, the output voltage of the charge amplifier in the high frequency region is simplified as:

(9) $$V_{o}=\frac{-V_{s}\;C_{a}}{C_{f}}$$

In Equation (9), the product of *V_s_* and *C_a_* is the charge *q*, which accumulated on the electrodes of the PVDF sensor, that is:

(10) $$q=V_{s}\;C_{a}$$

Thus, the output voltage in the high frequency region would be:

(11) $$V_{o}=\frac{-q}{C_{f}}$$

Equation (11) indicates that when the gain of the charge amplifier is large enough, the output voltage of the charge amplifier is not related to the gain of the charge amplifier but related to the charges accumulated on the PVDF sensor and the feedback capacitance of the charge amplifier.

However, in the low-frequency region, the term $\frac{1}{R_{f}}$ cannot be neglected. Therefore, the amplitude of output voltage of charge amplifier is expressed as:

(12) $$\left|V_{o}\right|=\frac{\omega q}{\sqrt{\frac{1}{R_{f}^{2}}+\omega^{2}\;C_{f}^{2}}}$$

If $\omega C_{f}=\frac{1}{R_{f}}$, the amplitude becomes:

(13) $$\left|V_{o}\right|=\frac{q}{C_{f}\;\sqrt{2}}$$

From Equation (12) and Equation (13), the frequency *ω* represents the cut-off frequency. The cut-off frequency in hertz is expressed as:

(14) $$f_{c}=\frac{1}{2\pi R_{f}\;C_{f}}$$

The phase of the output voltage in the low frequency region is expressed as:

(15) $$\phi =180{}^{\circ}+{\text{tan}}^{-1}\left(\frac{1}{\omega R_{f}\;C_{f}}\right)$$

It was noted that with the conditioning process of the charge amplifier, the cut-off frequency and the phase of measured voltage signal are both related to the feedback parameters of the charge amplifier. Therefore, we can moderate these parameters of the charge amplifier to minimize the loading effect in our measuring frequency region.

### 2.2. Experimental Setup of the PVDF Sensing System

**Figure 6.**
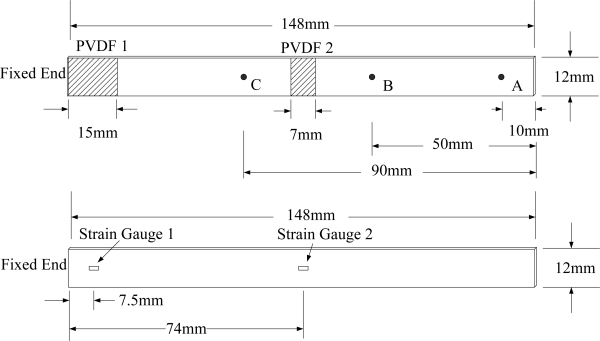
The locations of PVDF sensors and impact points on the upper surface, and the locations of strain gauges on the lower surface of the beam.

To study the accuracy of the dynamic strain measurement obtained with a PVDF film sensor, we used a conventional strain gauge, which has 1-mm gauge length, to measure the transient response of a cantilever beam simultaneously. The cantilever beam was 148 mm in length, 12 mm in width, and 1.6 mm in thickness. The beam was made of aluminum 1,050; its density was 2,705 Kg/m^3^. Young’s modulus was 69 GPa, Poisson’s ratio was 0.33, and the shear modulus was 25.8 GPa. The transient responses of the cantilever beam were induced by a freely dropped steel ball. Three impact locations were selected to excite transient responses of the cantilever beam. Point A was located 10 mm from the free end of the beam, point B was located 50 mm from the free end, and point C was located 90 mm from the free end. In addition, to study the effect of the size of the PVDF film sensor, two PVDF sensors with different sizes were used to measure transient responses. The first one was a 15-mm long PVDF sensor, which was bonded on the upper surface of the beam near the fixed end, while a strain gauge was bonded on the lower surface of the beam at the central point of the 15-mm PVDF sensor. The second one was a 7-mm long PVDF sensor, which was bonded on the upper surface at the center of the beam, while a strain gauge was bonded on the opposite surface of the central point of the 7-mm PVDF sensor. An illustration of the cantilever beam, the sensors, and impact locations is shown in Figure 6.

**Figure 7.**
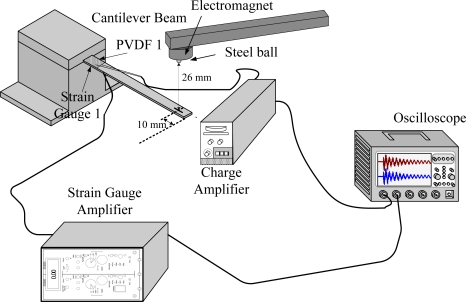
Experimental setup for two sensors to measure the transient response simultaneously.

Figure 7 shows the experimental setup of the measurement system. The steel ball was initially stuck by an electromagnet, and then it dropped freely from a height of 26 mm to the cantilever beam surface. The transient responses were measured by PVDF and strain gauge sensors simultaneously. Moreover, to study the influence of the charge amplifier, all measurements of the PVDF sensors were repeated twice. One was measured with the charge amplifier, while the other was not.

## 3. Theoretical Results of the Vibration Analysis for a Cantilever Beam

### 3.1. Resonant Frequencies of Bending and Lateral Modes of the Cantilever Beam

From the dimensions of the cantilever beam, we can see that the length is ten-fold larger than the width, and the width is ten-fold larger than the thickness. Hence, the theory of Bernoulli-Euler beam can be applied to analyze the resonant frequency of the cantilever beam [36]. The governing equation of motion of the Bernoulli-Euler beam is expressed as:

(16) $$\frac{\partial^{4}y}{\partial x^{4}}+\frac{1}{a^{2}}\;\frac{\partial^{2}y}{\partial t^{2}}=0$$

where *y* represents the transverse displacement of the beam, and:

(17) $$a^{2}=\frac{\mathrm{EI}}{\rho A}$$

in which *E* is Young’s modulus, *ρ* is the density, *A* is the cross-sectional area of the cantilever beam, and *I* is the moment of inertia. The frequency equation of the cantilever beam is expressed as follows:

(18) cos βℓ cosh βℓ=−1

where *ℓ* is the length of the beam. The first six roots of Equation (18) are:

$$\begin{array}{cccccc} \beta_{1}\ell =1.875, & \beta_{2}\ell =4.694, & \beta_{3}\ell =7.855, & \beta_{4}\ell =10.996, & \beta_{5}\ell =14.137, & \beta_{6}\ell =17.279 \end{array}$$

From the geometrical dimensions and material properties of the cantilever beam, the natural frequencies of bending modes of the cantilever beam can be evaluated. In addition, by exchanging the width *b* and thickness *h* of the cantilever beam, we can get the natural frequencies of lateral modes of the cantilever beam [37]. The exact analytical solution of a cantilevered piezoelectrical energy harvester with Bernoulli-Euler beam theory was presented [38].

### 3.2. Resonant Frequencies of Torsional Modes of the Cantilever Beam

The equation of motion of a cantilever beam for torsional modes is [39]:

(19) $$C_{T}\;\frac{\partial^{2}\theta }{\partial x^{2}}=\rho J\;\frac{\partial^{2}\theta }{\partial t^{2}}$$

where *θ* describes the angle of twist, *C_T_* is the torsional stiffness, and *J* is the polar area moment of inertia. In the case that the width of the beam is much larger than the thickness, *J* can be expressed as:

(20) $$J=\frac{c^{3}\;b}{12}$$

where *c* is the width of the beam and *b* is the thickness of the beam. The torsional stiffness, *C_T_*, is expressed as:

(21) $$C_{T}=\frac{{\mathrm{cb}}^{3}}{3}\;G$$

where *G* is the shear modulus of the cantilever beam. The resonant frequency can be explicitly expressed as:

(22) $$f_{n}=\frac{\left(2n+1\right)}{2\ell }\;\frac{b}{c}\;\sqrt{\frac{G}{\rho }}$$

## 4. Experimental Results

Figure 6 shows the geometrical dimension and locations of PVDF films and strain gauges. Figure 7 illustrates the experimental setup of the dynamic measurement system. The two sensors measured the transient response of dynamic strain for the cantilever beam simultaneously. All of the results that were measured by a PVDF sensor were compared with those obtained with a strain gauge. The transient responses of the cantilever beam were excited by a freely dropped steel ball from three different locations on the beam. The diameter of the steel ball was 3.17 mm, and the steel ball was dropped from 26 mm above the cantilever beam.

In the first part of the experiment, the first pair of sensors measured transient responses of the beam subjected to three different impact locations. In addition, the steel ball was dropped twice at each impact location. For the first transient response, the PVDF sensor measured the signal with a charge amplifier. However, for the second transient response, the PVDF sensor measured the signal without the charge amplifier. Similarly, in the second part of the experiments, the second pair of sensors measured transient responses for three impact locations.

### 4.1. The Measured Results for the First Pair of Sensors

**Figure 8.**
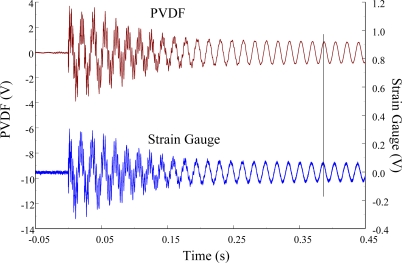
The transient responses within 450 ms of the first pair of sensors for impact location A (with the charge amplifier).

**Figure 9.**
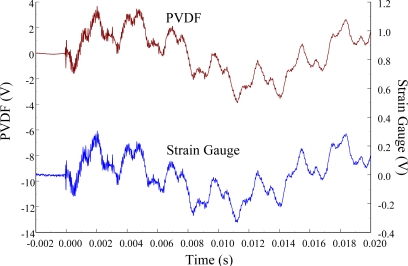
The transient responses within 20 ms of the first pair of sensors for impact location A.

The experimental setup is shown in Figure 7. The transient responses, which were excited by the impact of a steel ball, were measured by a PVDF sensor and a strain gauge simultaneously. The signals, which were measured by the PVDF sensor, were input to the charge amplifier and then to the oscilloscope. On the other hand, signals measured by the strain gauge passed through a Wheatstone bridge and a gauge amplifier and then shown in the oscilloscope. The PVDF sensor and the strain gauge were bonded on the surfaces near the fixed end of the cantilever beam. The size of the PVDF sensor was 15 mm × 12 mm × 28 μm, and the gauge length of strain gauge was 1 mm. The locations of the PVDF sensor, the strain gauge, and the impact points on the cantilever beam are shown in Figure 6. For impact location A, the measured result, where the PVDF sensor measures with the charge amplifier, within 450 ms is shown in Figure 8. There were 2.5 million measuring points in each measurement. The measured results within 20 ms are shown in Figure 9. There were 110 thousand points in each measurement. Good agreement between the measured results was found with the PVDF sensor and the strain gauge. Figure 8 and Figure 9 illustrate the relative large amplitude of background noise in the strain gauge and the much higher signal-to-noise ratio of the PVDF sensor. Therefore, at low strain levels, the signal to noise ratio of strain gauges was poor. The superior signal to noise ratio of PVDF sensors makes them much more attractive in situations of low strain or high noise level.

**Figure 10.**
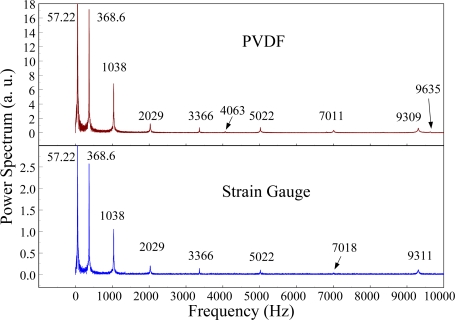
The frequency spectrum of the first pair of sensors for impact location A (with the charge amplifier).

The Fast Fourier Transformation was used to transfer the transient responses from the time domain to the frequency domain. The frequency spectra of the PVDF sensor and the strain gauge within 10 kHz are shown in Figure 10. Table 1 shows the resonant frequencies and the correspondent mode shapes predicted by FEM. The locations of the impact points and the sensors are also indicated in Table 1. As shown in Table 1, impact location A was near the nodal line of the higher bending mode (*i.e*., from mode 4 to mode 10) and close to the nodal lines of the torsional modes (*i.e*., modes 6, 8, 11) of the beam. Hence, the magnitude of the resonant frequency for the frequency spectrum was relatively low from mode 4 to mode 11. Additionally, the location of the strain gauge was at the nodal lines of all of the torsional modes. Therefore, the energies of resonant torsional modes at 4063 Hz, 7011 Hz and 9635 Hz were too small for the strain gauge to measure. However, the PVDF sensor was sensitive enough to measure all bending modes and two torsional modes.

**Figure 11.**
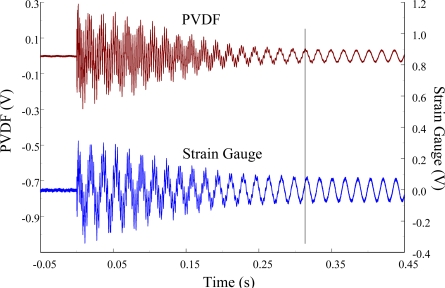
The transient responses within 450 ms of the first pair of sensors for impact location A (without the charge amplifier).

**Figure 12.**
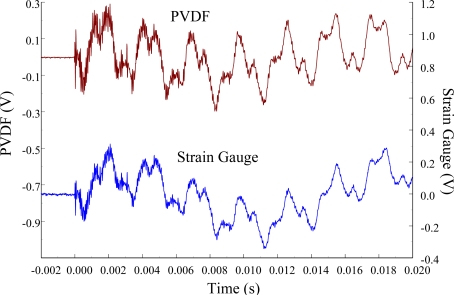
The transient responses within 20 ms of the first pair of sensors for impact location A.

**Figure 13.**
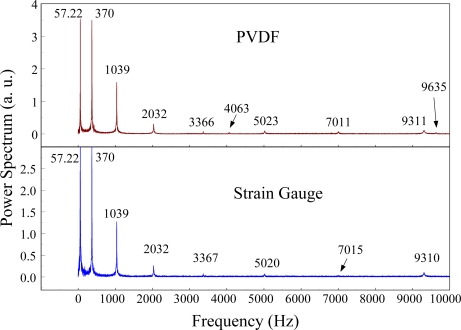
The frequency spectrum of the first pair of sensors for impact location A.

Figures 11 and 12 show the results of the PVDF sensor and the strain gauge within 450 ms and 20 ms, respectively, in the case of the PVDF sensor in the absence of the charge amplifier. The signal in the time domain measured by the PVDF sensor without the charge amplifier was different from that obtained with the strain gauge. In addition, because of the capacitance of the PVDF sensor, there was a phase shift of the first bending mode. Based on data in Figure 8 (Figure 11) and Figure 9 (Figure 12), we can see that the transient responses measured by the strain gauge for the two repeated experiments were nearly identical. By applying the Fast Fourier Transform to the measured results of the PVDF sensor and the strain gauge in the time-domain, the frequency spectrum within 10 kHz was obtained, as shown in Figure 13. Because of the high-pass filter characteristics of the PVDF sensor, the energy of the first bending mode measured by the PVDF sensor was lower than expected. The high-pass filter characteristics influence the first bending mode most strongly because the cut-off frequency, 115 Hz, is between the first resonant frequency and the second resonant frequency. Although there was a discrepancy between the transient responses measured by the PVDF sensor and the strain gauge, the data in Figure 13 indicate that the resonant frequencies were accurately determined by the PVDF sensor without the charge amplifier.

**Figure 14.**
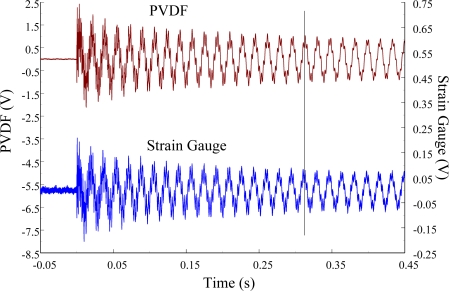
The transient responses within 450 ms of the first pair of sensors for impact location B (with the charge amplifier).

**Figure 15.**
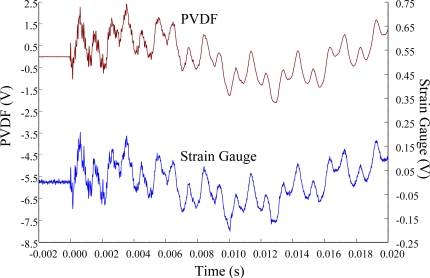
The transient responses within 20 ms of the first pair of sensors for impact location B.

**Figure 16.**
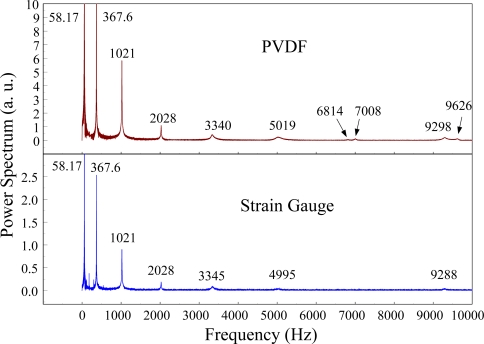
The frequency spectrum of the first pair of sensors for impact location B.

At impact location B, which was 50 mm from the free end of the cantilever beam, the measured results within 450 ms and 20 ms for the PVDF sensor with the charge amplifier and the strain gauge were obtained, as shown in Figure 14 and Figure 15. We can see that there was no phase shift between the signals measured by the PVDF sensor and those measured by the strain gauge. The measured results within 10 kHz in the frequency domain are shown in Figure 16. Again, the torsional modes could not be measured by the strain gauge, but they could be obtained with the PVDF sensor. On the other hand, referring to Table 1, impact location B was near the two nodal lines of the second torsional mode, 4,063 Hz, thus the frequency spectrum of the PVDF sensor did not show this mode.

**Figure 17.**
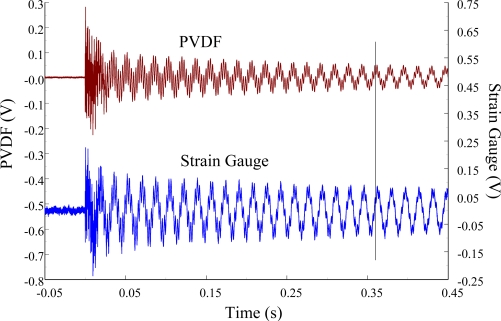
The transient responses within 450 ms of the first pair of sensors for impact location B (without the charge amplifier).

**Figure 18.**
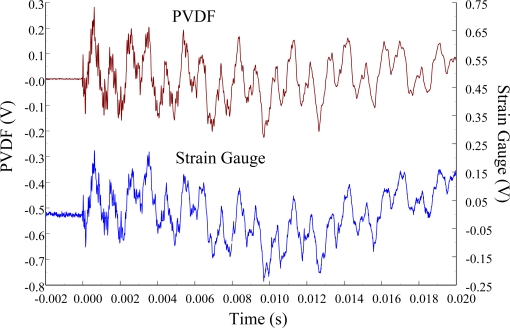
The transient responses within 20 ms of the first pair of sensors for impact location B.

**Figure 19.**
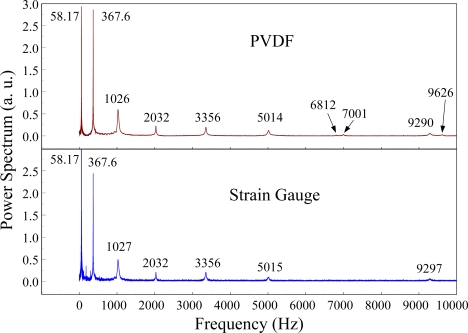
The frequency spectrum of the first pair of sensors for impact location B.

In addition, in order to see the influence of the charge amplifier, the PVDF sensor was used without the charge amplifier. The transient results of the PVDF sensor and the strain gauge within 450 ms and 20 ms are shown in Figures 17 and 18. Without the compensation of the charge amplifier, these two signals had different waveforms, and there was a phase shift between them. The frequency spectra of the transient responses measured by the PVDF sensor and by the strain gauge within 10 kHz are shown in Figure 19.

**Figure 20.**
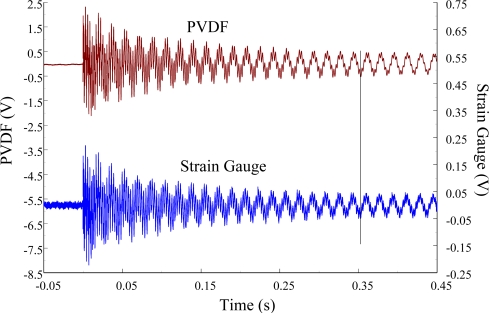
The transient responses within 450 ms of the first pair of sensors for impact location C (with the charge amplifier).

**Figure 21.**
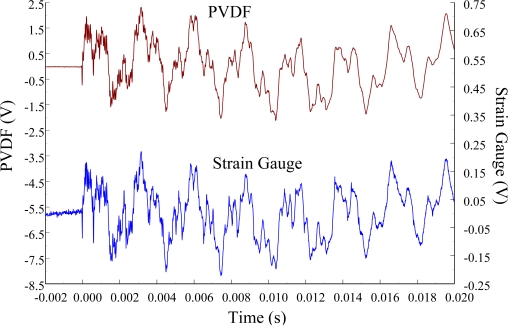
The transient responses within 20 ms of the first pair of sensors for impact location C.

**Figure 22.**
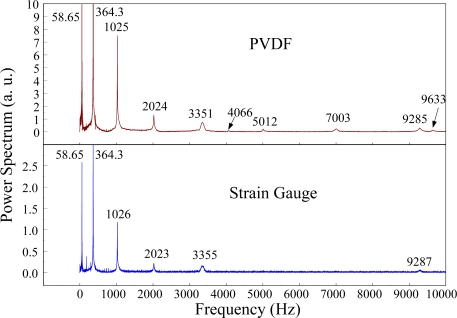
The frequency spectrum of the first pair of sensors for impact location C.

For impact location C, which was 90 mm from the free end of the cantilever beam, the measured results of the PVDF sensor and the strain gauge are shown in Figures 20 and 21. Good agreements were found between the transient results of these two sensors. In the frequency domain, the measured results of the PVDF sensor and the strain gauge within 10 kHz are shown in Figure 22. Because impact location C was near two nodal lines of the third torsional mode, 6,714 Hz, and the nodal lines of the sixth and the seventh bending modes, the PVDF sensor could not measure the third torsional mode, and the strain gauge could not measure these two bending modes.

**Figure 23.**
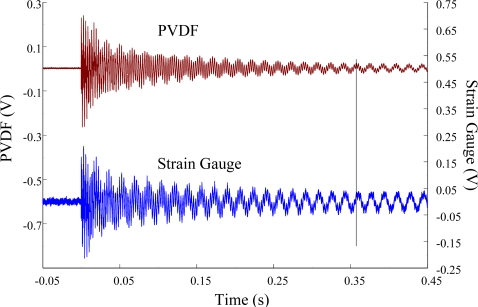
The transient responses within 450 ms of the first pair of sensors for impact location C (without the charge amplifier).

**Figure 24.**
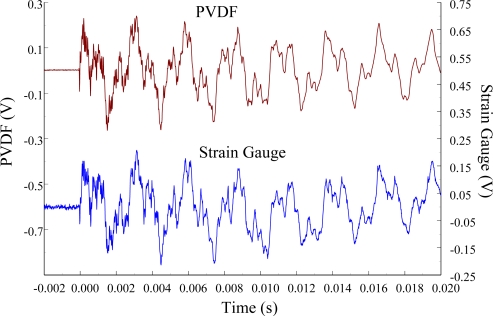
The transient responses within 20 ms of the first pair of sensors for impact location C.

**Figure 25.**
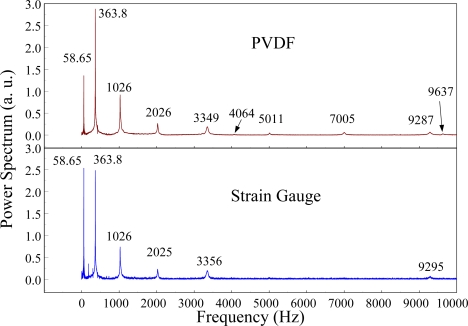
The frequency spectrum of the first pair of sensors for impact location C.

The measured results of the PVDF sensor without the charge amplifier and the strain gauge within 450 ms and 20 ms are shown in Figures 23 and 24, and these transient results in the frequency domain within 10 kHz are presented in Figure 25. Without the compensation of the charge amplifier, the energy of the first bending mode, when measured by the PVDF sensor, was much lower than expected. Therefore, the measured results of the dynamic strain in the time domain from the PVDF sensor and the strain gauge did not agree. However, the resonant frequencies determined with the PVDF sensor without the charge amplifier (*i.e*., Figure 25) are the same as those obtained with the PVDF sensor with the charge amplifier (*i.e*., Figure 22).

### 4.2. The Measured Results for the Second Pair of Sensors

**Figure 26.**
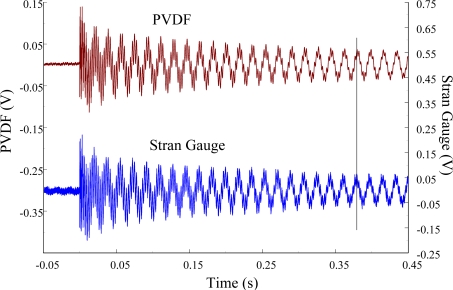
The transient responses within 450 ms of the second pair of sensors for impact location A (with the charge amplifier).

**Figure 27.**
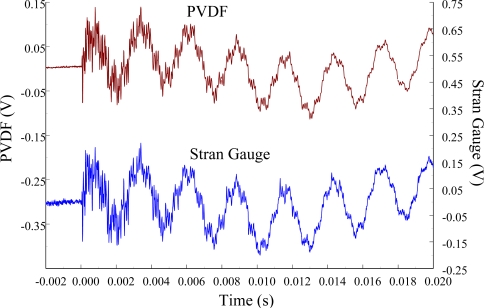
The transient responses within 20 ms of the second pair of sensors for impact location A.

In this section, we used the second PVDF sensor that is half the size of the first one to measure the transient responses of the beam. In the second pair of sensors, the size of the PVDF sensor was reduced to 7 mm × 12 mm × 28 μm. This pair of sensors was located on the middle point of the beam as shown in Figure 26. The experimental setup was the same as that of the first pair of sensors as shown in Figure 27. The transient responses at the middle point of the beam for impact locations A, B, and C are presented.

At impact location A, the measured results of the PVDF sensor with the charge amplifier and the strain gauge within 450 ms and 20 ms in time domain were obtained, as shown in Figures 26 and 27, respectively. Good agreement was found between the results of the PVDF sensor and the strain gauge. Although the size of the second PVDF sensor was half that of the first one, the ability to measure the transient response was the same for two PVDF sensors when the charge amplifier was used.

**Figure 28.**
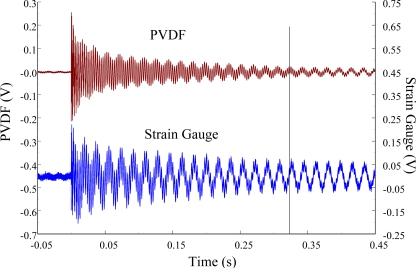
The transient responses within 450 ms of the second pair of sensors for impact location A (without the charge amplifier).

**Figure 29.**
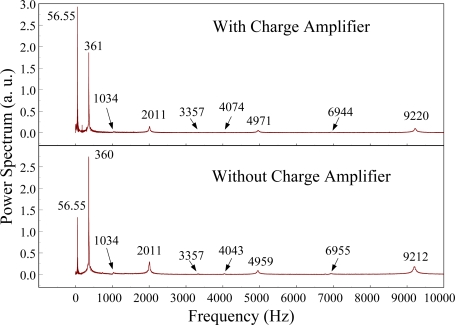
The frequency spectrum of the second PVDF sensor with and without the charge amplifier for impact location A.

The PVDF sensor was also used without the charge amplifier, and the measured result of the PVDF sensor within 450 ms is shown in Figure 28. From Equation (1) and Equation (9), because the size of the second PVDF sensor is smaller than that of the first PVDF sensor, the cut-off frequency of this PVDF sensor was higher than that of the first one. Therefore, the high-pass filter characteristic of the second PVDF sensor influenced the measured results in the low frequency range more than the first PVDF sensor. The transient results of the PVDF sensors with and without the charge amplifier in the frequency domain are shown in Figure 29. It is indicated in this figure that, due to the high-pass filter characteristic of the second PVDF sensor without the charge amplifier, the energy of the first bending mode was much lower than expected. Although there were differences between the transient results of the PVDF sensors with and without the charge amplifier, the values of the resonant frequencies in these two cases were nearly identical. Therefore, the size of the PVDF sensor may influence the measurement of low-frequency transient responses if the charge amplifier was not used, yet the resonant frequency can be accurately determined.

**Figure 30.**
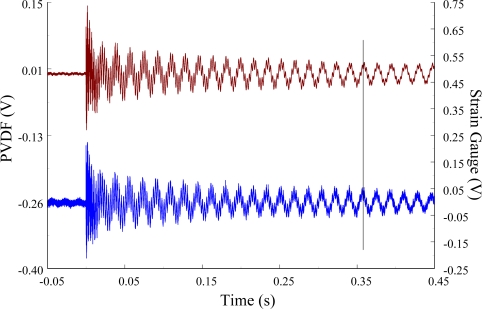
The transient responses within 450 ms of the second pair of sensors for impact location B (with the charge amplifier).

**Figure 31.**
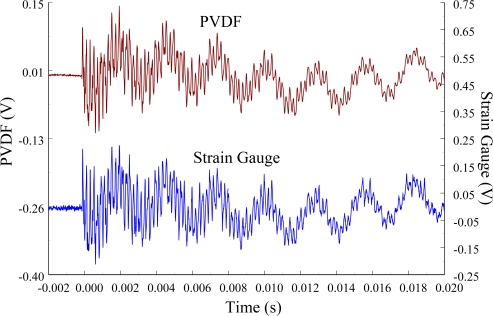
The transient responses within 20 ms of the second pair of sensors for impact location B.

**Figure 32.**
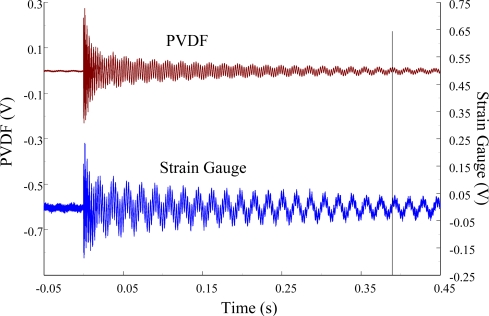
The transient responses within 450 ms of the second pair of sensors for impact location B (without the charge amplifier).

The measured results of the second pair of sensors within 450 ms and 20 ms in the time domain at impact location B are shown in Figure 30 and Figure 31, respectively. In these two figures, the PVDF sensors were measured with the charge amplifier. These results were consistent with each other, and there was no phase shift between the signals of these two sensors. The PVDF sensor was also used without the charge amplifier, and the measured result of the second pair of sensors is shown in Figure 32. From this figure, we can see that if the PVDF sensor was used without the charge amplifier, the result between the PVDF sensor and the strain gauge was significantly different, and there was a phase shift between these results.

**Figure 33.**
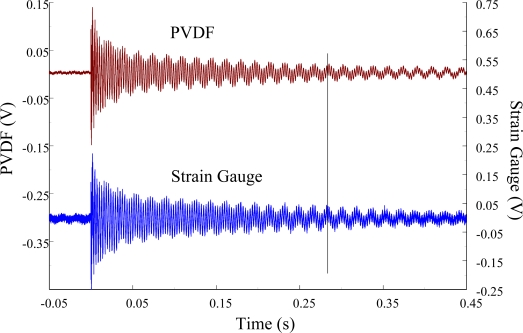
The transient responses within 450 ms of the second pair of sensors for impact location C (with the charge amplifier).

**Figure 34.**
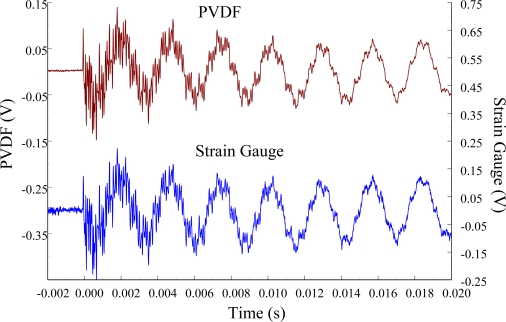
The transient responses within 20 ms of the second pair of sensors for impact location C.

**Figure 35.**
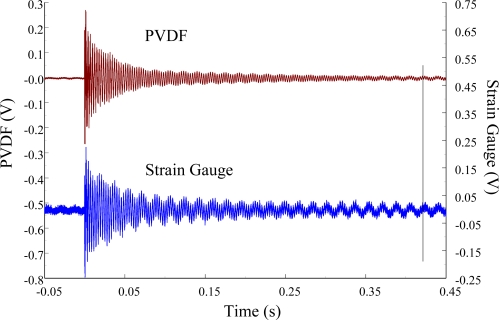
The transient responses within 450 ms of the second pair of sensors for impact location C (without the charge amplifier).

The measured results of the second pair of sensors within 450 ms and 20 ms in the time domain at impact point C are shown in Figures 33 and 34, respectively. In these two results, the PVDF sensors were used with the charge amplifier, and the measured results of the PVDF sensor and the strain gauge were in good agreement. The measured result of the PVDF sensor without the charge amplifier is shown in Figure 35. We can see that the measured results of the PVDF sensor and the strain gauge were quite different, and there was a phase shift between these two results.

To summarize, we averaged all of the resonant frequency values measured by two pairs of sensors at three impact locations, and the results are presented in Tables 2 and 3. These experimental measured data were compared with the ABAQUS finite element calculations and the theoretical results. Table 2 shows the measured results of the first pair of sensors, and Table 3 shows the second pair of sensors. The discrepancies in the measured frequencies from two pairs of sensors were less than 2.5% when compared with the theoretical results. Therefore, the accuracies of these two pairs of sensors were confirmed.

## 5. Conclusions

In this article, we studied the influence of the size of the PVDF film, nodal lines of the cantilever beam, and the use of the charge amplifier on sensing ability. All of the results, which were measured by a PVDF sensor, were compared with those obtained with a strain gauge in addition to theoretical calculations and FEM results. The accuracies of measuring the dynamic strain in a transient situation from two pairs of sensors were confirmed. Referring to the measured results of these two pairs of sensors, we can conclude that a charge amplifier is indispensable for a PVDF sensor, especially a small one, to improve the low-frequency responses of the measured results of the PVDF sensor. Moreover, referring to the measured results of the first pair of sensors, the strain gauge could not measure some bending modes and torsional modes. However, the PVDF sensor could measure almost all modes under the same conditions. Therefore, based on these results, it was concluded that the sensitivity of the PVDF sensor was superior to that of the strain gauge.

## Figures and Tables

**Table 1.** The mode shapes, the locations of sensors, and impact points.

**Mode 1: 59.2Hz (Bending Mode)**
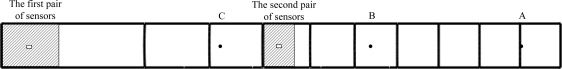
**Mode 2: 370Hz (Bending Mode)**
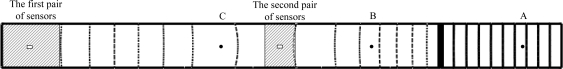
**Mode 3: 1038Hz (Bending Mode)**
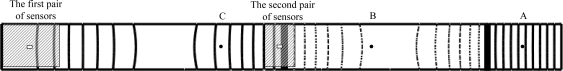
**Mode 4: 2033Hz (Bending Mode)**
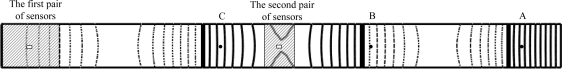
**Mode 5: 3361Hz (Bending Mode)**
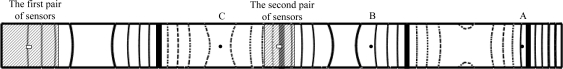
**Mode 6: 4017Hz (Torsional Mode)**
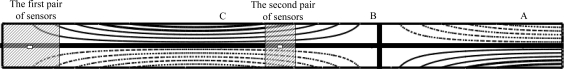
**Mode 7: 5019Hz (Bending Mode)**
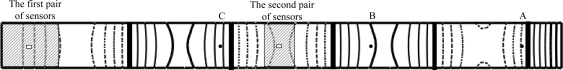
**Mode 8: 6714Hz (Torsional Mode)**
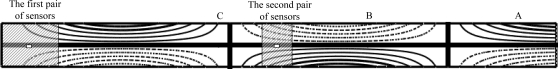
**Mode 9: 7007Hz (Bending Mode)**
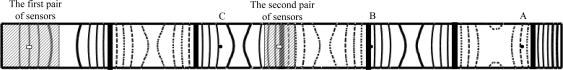
**Mode 10: 9321Hz (Bending Mode)**
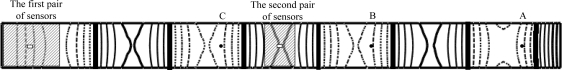
**Mode 11: 9533Hz (Torsional Mode)**
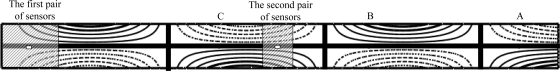

**Table 2.** The comparison of the resonant frequencies for the first pair of sensors, the ABAQUS, and the theory.

**Mode**		**Theory (Hz)**	**FEM (Error %)**	**PVDF (Error %)**	**Strain Gauge (Error %)**
1		58.84	59.2 (0.63)	58.01 (−1.4)	58.01 (−1.4)
2		368.8	370.8 (0.55)	367.3 (−0.4)	366.6 (−0.5)
3		1,032.8	1,038.2 (0.50)	1,028.3 (−0.4)	1,028.3 (−0.4)
4		2,023.8	2,033.5 (0.48)	2,028 (0.2)	2,026.6 (0.1)
5		3,345.2	3,361.1 (0.47)	3,352.3 (0.2)	3,355.3 (0.3)
6	Torsional	4,121.3	4,017(−2.5)	4,064.5(−1.3)	
7		4,997.4	5,019.3 (0.44)	5,018 (0.4)	5,010 (0.2)
8	Torsional	6,868.8	6,714 (−2.2)	6,814 (−0.8)	
9		6,979.6	7,007.2 (0.39)	7,007.3 (0.4)	7,018 (0.6)
10		9,292.3	9,321.5 (0.31)	9,297.3 (0.05)	9,295.3 (0.03)
11	Torsional	9,616.3	9,533 (−0.8)	9,631.3 (0.15)	

**Table 3.** The comparison of the resonant frequencies for the second pair of sensors, the ABAQUS, and the theory.

**Mode**		**Theory (Hz)**	**FEM (Error %)**	**PVDF (Error %)**	**Strain Gauge (Error %)**
1		58.84	59.2 (0.63)	56.52 (−2.5)	56.52 (−2.5)
2		368.8	3,70.8 (0.55)	361.1 (−2.0)	361.1 (−2.0)
3		1,032.8	1,038.2 (0.50)	1030 (−0.2)	
4		2,023.8	2,033.5 (0.48)	2,010.3 (−0.6)	2,009 (−0.7)
5		3,345.2	3,361.1 (0.47)	3,336.3 (−0.2)	
6	Torsional	4,121.3	4,017(−2.5)	4,045.5 (−1.8)	
7		4,997.4	5,019.3 (0.44)	4,949.3 (−0.9)	4,949.6 (−0.9)
8	Torsional	6,868.8	6,714 (−2.2)		
9		6,979.6	7,007.2 (0.39)	6,942 (−0.5)	
10		9,292.3	9,321.5 (0.31)	9,205.3 (−0.9)	9,206.6 (−0.9)
11	Torsional	9,616.3	9,533 (−0.8)		
